# Comparison of three radiographic assessment methods for detecting slipped capital femoral epiphyses in cats: Klein’s line, modified Klein’s line and the S-sign

**DOI:** 10.1177/1098612X231201775

**Published:** 2023-10-31

**Authors:** Daniel Butts, Alex J Smith, Kate Bradley, Richard Meeson, Kevin Parsons, Sorrel J Langley-Hobbs

**Affiliations:** 1 Stonehouse Veterinary Practice, Gloucester, UK; 2 Highcroft Veterinary Referrals, Diagnostic Imaging, Bristol, UK; 3 University of Bristol Veterinary School, Faculty of Health Sciences, Bristol, UK; 4 The Royal Veterinary College Department of Clinical Science and Services, Hatfield, Hertfordshire, UK; 5 Langford Vets, University of Bristol Veterinary School, Faculty of Health Sciences, Bristol, UK

**Keywords:** Femur, fracture, imaging, castrated, pathological, slipped capital femoral epiphyses

## Abstract

**Objectives:**

The aim of the present study was to investigate whether diagnostic assessment methods used on radiographs in humans with slipped capital femoral epiphysis (SCFE) can be used in cats.

**Methods:**

The ventrodorsal (VD) extended-leg and VD frog-leg pelvic radiographs of 20 cats with SCFE without fully displaced femoral capital epiphyses (FCE), eight cats with fully displaced FCE and five control cats with normal pelvic anatomy were assessed by five observers on two separate occasions 3 months apart. The Klein’s line and modified Klein’s line were assessed on each VD extended-leg radiograph, and the S-sign was assessed on each VD extended-leg and VD frog-leg radiograph.

**Results:**

Excluding cases of fully displaced FCE, the S-sign on the VD frog-leg radiographs more accurately diagnosed SCFE than the S-sign on the VD extended-leg radiographs and the Klein’s line (92.4% vs 88.8% vs 60.6%, respectively), and had the greatest sensitivity (93.9% vs 79.2% vs 30.6%, respectively). The S-sign on the VD extended-leg radiographs had greater specificity than the Klein’s line and S-sign on the VD frog-leg radiographs (99.2% vs 97.9% vs 90.9%, respectively). The modified Klein’s line detected SCFE in 40.2% of cases that were negative for the Klein’s line.

**Conclusions and relevance:**

The S-sign in both VD extended-leg and VD frog-leg views successfully detected SCFE in cats and can be used to increase early diagnosis and treatment in cats with SCFE that have only subtle radiographic changes.

## Introduction

Slipped capital femoral epiphysis (SCFE) has previously been described in cats as a progressive condition resulting in displacement of the proximal femoral epiphysis, which occurs due to abnormalities of the proximal femoral physis.^1–5^ Histologically, characteristic changes include a widened physis with irregular clusters of chondrocytes within an abundant extracellular matrix.^1–4^ Affected cats present with either a unilateral or bilateral hindlimb lameness in the absence of trauma^1,5^ and may suffer spontaneous femoral capital physeal fracture.^2,6^ A strong male predisposition has been identified and almost all affected cats are neutered.^1–5^ It has been suggested that delayed physeal closure may be a predisposing factor.^1,2,4^ Prepubertal neutering in cats has been associated with delayed physeal closure.^7,8^ Although frequently reported in domestic shorthairs (DSHs), it also appears that Maine Coon and Siamese cats are predisposed to the condition.^1–3^ Typical radiographic features include an open proximal femoral physis, osteolysis and sclerosis of the femoral neck, and displacement of the proximal femoral epiphysis.^1,2,6,9^ Previous reports have also labelled the condition femoral neck metaphyseal osteopathy and spontaneous capital femoral physeal fracture,^5,6^ but it is believed these are in fact the same condition and SCFE has become the accepted terminology for the disease. In previous literature, the most commonly reported treatment option for cats with SCFE is a femoral head and neck ostectomy.^1,2,5^ Surgical reduction and stabilisation with K-wires has also been reported, with one study highlighting a short recovery and good prognosis for return to normal function in these cats.^1,2,10^ More recently, treatment with a total hip replacement has been reported.^1^ Although rarely described, conservative management is a further option.^2^

SCFE is the most common hip abnormality in adolescent humans.^11,12^ Early diagnosis is extremely important to allow prompt treatment to optimise long-term functional outcome.^11,13^ However, subtle and early abnormalities are often missed on radiographs.^11,14,15^ Because of the difficulties in early diagnosis in humans, a variety of radiographic assessment methods have been developed to aid in its identification. These include the Klein’s line, modified Klein’s line and the S-sign.^11,16–19^

The Klein’s line is a tangent drawn on the lateral margin of the femoral neck on an anteroposterior (AP) pelvic radiograph (equivalent to a ventrodorsal [VD] extended-leg pelvic view). A positive result is when the line does not intersect the epiphysis.^11,16,17^

Owing to poor sensitivity of the Klein’s line in cases of mild epiphyseal displacement, the modified Klein’s line method was developed. This involves a Klein’s line being drawn bilaterally and the amount of epiphyseal intersection measured. A positive result is one where the affected side has reduction in epiphyseal intersection >2 mm in comparison to the unaffected limb.^16–18^

A further method to improve diagnostic sensitivity is the S-sign. This is measured on a frog-leg lateral pelvic radiograph and is a curvilinear line drawn from the lesser trochanter, continuing along the femoral neck, across the line of the physis and wrapping around the femoral head to the midpoint. A broken continuity, asymmetry or sharp turn is a positive result.^19^

Although advanced stages of SCFE in cats are typically easy to identify on radiographs, subtle changes early in the course of the disease are harder to detect,^9^ especially in cases of acute fracture with minimal displacement or in those with chronic fractures.^2^ Early diagnosis of subtle SCFE could lead to earlier surgical treatment^9^ and avoid the need for salvage surgery, such as femoral head and neck excision or total hip replacement.

Application of these human radiographic assessment methods to the radiographs of cats with SCFE has not been published. The objectives of this study were to apply the Klein’s line, modified Klein’s line and the S-sign to the radiographs of cats with SCFE. We aimed to investigate whether these methods can be used successfully to aid in the diagnosis of cats with SCFE. We hypothesised that the anatomical differences between humans and cats would reduce the effectiveness of the Klein’s line and the modified Klein’s line, and that the S-sign would be the most effective of these methods for the detection of SCFE in cats.

## Materials and methods

This study was a retrospective case-control study. Ethical approval was granted by the Animal Welfare and Ethical Review Body, University of Bristol (reference VIN/20/020).

Clinical records from Langford Small Animal Hospital and The Queen Mother Hospital for Animals were searched for cats with pelvic radiographs between 1 January 2010 and 14 May 2020. Cats were included in the study if they had been diagnosed with SCFE without full displacement of their FCE, and had both VD extended-leg and VD frog-leg pelvic radiographs available. A total of 20 cats met the inclusion criteria. Eight cats with unilateral fully displaced FCE were included as a control group, and a further five cats with normal VD extended-leg and VD frog-leg pelvic radiographs were included as an unaffected control group.

**Figure 1:**
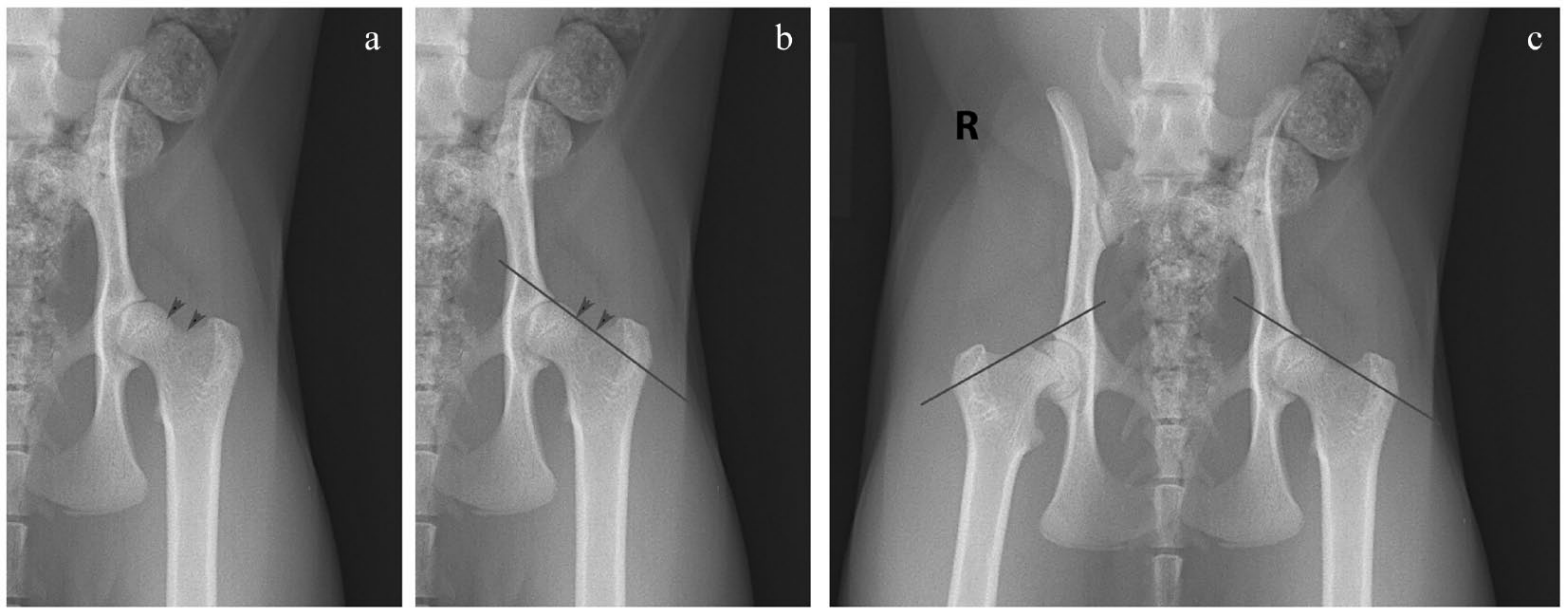
The Klein’s line. Ventrodorsal extended-leg view. (a) The arrowheads indicate the proximal and distal aspects of the lateral margin of the femoral neck. (b) The Klein’s line is drawn at the tangent of the curve between these two arrowheads and it intersects the femoral capital epiphysis – this is a negative result. (c) The Klein’s line does not intersect the femoral capital epiphysis on the right side and so this is a positive result indicating displacement of the epiphysis. The left side is negative

For all 33 cases, the lateral margins of the femoral necks were identified on the VD extended-leg images. Owing to the concavity of this lateral margin, the proximal and distal aspects of the femoral neck were identified. The Klein’s line was drawn at the tangent of this curvature. A positive result occurred when the line did not intersect the epiphysis and would be indicative of a femoral capital epiphyseal slip (Figure 1).

**Figure 2:**
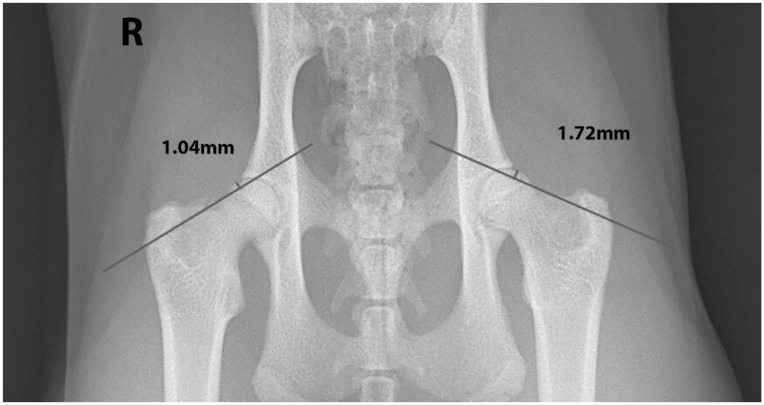
The modified Klein’s line. Ventrodorsal extended-leg view. In this example, 1.72 mm of the epiphysis is intersected by the Klein’s line on the left side, and 1.04 mm of the epiphysis is intersected by the Klein’s line on the right side. The difference between the two sides is 0.68 mm. In this study, we have defined that a difference of 0.5 mm or greater between the two sides is indicative of a positive result. Therefore, in this example, a positive result of epiphyseal displacement would be given to the right side

For cases without a positive result for the Klein’s line, the modified Klein’s line was performed. For these cases, the Klein’s line was drawn on both sides and the amount of femoral capital epiphyseal intersection was calculated on each side. Owing to the much smaller size of cats compared with humans, the difference of >2 mm required for a positive in humans was too high; therefore, for the purpose of this study, a positive result was determined when the difference in epiphyseal intersection between the two sides was ⩾0.5 mm. The side with the least intersection was given a positive result indicative of femoral capital epiphyseal displacement (Figure 2).

**Figure 3:**
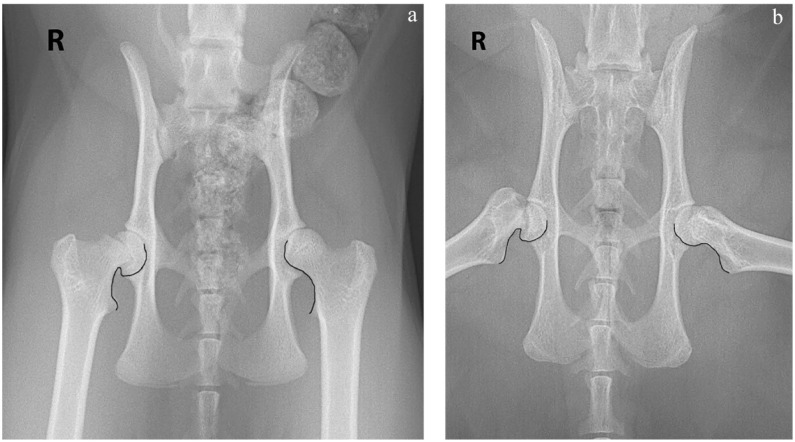
The S-sign. (a) Ventrodorsal (VD) extended-leg view. A curvilinear line is drawn from the lesser trochanter, along the femoral neck and around the femoral head to its midpoint. The left side on this image is normal. The right side illustrates a sharp turn and so is indicative of an abnormal result. (b) VD frog-leg view. The curvilinear line is drawn in the same way as on the VD extended-leg view. The left side on this image is normal. The right side illustrates a sharp turn and so is indicative of an abnormal result

For the purpose of this study, the S-sign was measured on both VD extended-leg and VD frog-leg images in all 33 cases. For each view, a curvilinear line was drawn from the lesser trochanter, continuing along the femoral neck, across the line of the physis and wrapping around the femoral head to the midpoint. A broken continuity, asymmetry or sharp turn was indicative of a positive result for femoral capital epiphyseal displacement (Figure 3).

Images were analysed using an open-source medical image viewer software (Horos [Horosproject.org], sponsored by Purview). The VD extended-leg and VD frog-leg pelvic radiographs for all 33 cases were de-identified and case order randomly arranged. Five observers reviewed each of the radiographs on two separate occasions 3 months apart. The observers were instructed on how to draw and interpret the Klein’s line, modified Klein’s line and the S-sign. The observers included two European specialists in veterinary diagnostic imaging and three European specialists in small animal surgery.

The accuracy, sensitivity, specificity, positive predictive value and negative predictive value were reported for each parameter. For the modified Klein’s line, only unilateral cases, and those cases in which the observer determined both sides to be negative for the Klein’s line, were included in the analysis. The intra- and inter-observer reliabilities of the Klein’s line, modified Klein’s line and S-sign were measured using the Cohen’s kappa coefficient. The relative strength of agreement of the kappa statistics were evaluated according to Landis and Koch: <0.00 = poor; 0.00–0.20 = slight; 0.21–0.40 = fair; 0.41–0.60 = moderate; 0.61–0.80 = substantial; and 0.81–1.00 = almost perfect.^20^

## Results

In total, 20 cats (19 males, one female, all neutered) with SCFE without fully displaced FCE were included in the study. Of these affected cats, there were six DSHs, four Maine Coons, five British Shorthairs, three Bengals, one domestic longhair and one British Blue. The age range of the cats was 7–44 months (mean age 20.8 ± 8.2 months; median 20 months).

Six cats presented with bilateral SCFE, seven with left-sided SCFE and seven with right-sided SCFE. Of the bilateral cases, four had moderate displacement of the femoral capital physis bilaterally, and two had one side with mild displacement and the other with moderate displacement. Of the remaining unilateral cases, 10 had mild displacement, one had moderate displacement and three had severe displacement. An additional eight cats with fully displaced FCE and five cats with normal pelvic anatomy were included as control groups. Determination of mild, moderate and severe displacement was subjective and based on consensus agreement between the authors.

When all 33 cases were included in the analysis (including the fully displaced FCE control group), the Klein’s line was able to identify SCFE with a mean accuracy of 69.6% (median 68.2%), sensitivity of 44.1% (median 41.2%), specificity of 96.5% (median 98.5%), positive predictive value of 92.5% (median 97.6%) and negative predictive value of 62.3% (median 60.6%).

The modified Klein’s line was only performed in unilateral cases that did not produce a positive result for the Klein’s line. This is because this method is not to be used in bilaterally affected cases and can only be used when both sides produce a negative result for the Klein’s line. The mean number of cases for which the modified Klein’s line was applied on each round was 18 (range 14–23 for round 1 and 12–21 for round 2). Of these cases, the modified Klein’s line yielded a mean accuracy of 50.3% (median 53.4%), sensitivity of 41.2% (median 49.5%), specificity of 65.1% (median 66.7%), positive predictive value of 59.6% (median 66.7%) and negative predictive value of 32.1% (median 35.4%). The S-sign performed in the VD extended-leg position produced a mean accuracy of 89.4% (median 90.9%), sensitivity of 81.8% (median 82.4), specificity of 97.5% (median 96.9%), positive predictive value of 97.2% (median 96.9%) and negative predictive value of 83.9% (median 84.2%). When the S-sign was performed in the VD frog-leg position, the mean accuracy was 90.5% (median 90.9%), sensitivity of 91.5% (median 95.6%), specificity of 89.4% (median 89.1%), positive predictive value of 90.1% (median 90.3%) and negative predictive value of 91.2% (median 94.9%). These results are summarised in Table 1. The mean results from each round of readings for each of the observers have also been provided in Table 2.

**Table 1:** The accuracy, sensitivity, specificity, positive predictive value and negative predictive value for the Klein’s line, modified Klein’s line and S-sign in the ventrodorsal (VD) extended-leg position and the S-sign in the VD frog-leg position when the fully displaced femoral capital epiphyses control group was included in the analysis

	Klein’s line	Modified Klein’s line	S-sign VD extended-leg	S-sign VD frog-leg
Accuracy (%)				
Mean	69.6	50.3	89.4	90.5
Median	68.2	53.4	90.9	90.9
Sensitivity (%)				
Mean	44.1	41.2	81.8	91.5
Median	41.2	49.5	82.4	95.6
Specificity (%)				
Mean	96.5	65.1	97.5	89.4
Median	98.5	66.7	96.9	89.1
Positive predictive value (%)				
Mean	92.5	59.6	97.2	90.1
Median	97.6	66.7	96.9	90.3
Negative predicitive value (%)				
Mean	62.3	32.1	83.9	91.2
Median	60.6	35.4	84.2	94.9

**Table 2:** The mean results from each round of readings from each of the observers when the fully displaced femoral capital epiphyses control group was included in analysis

	Klein’s line		Modified Klein’s line		S-sign extended		S-sign frog-leg	
	Round 1	Round 2	Round 1	Round 2	Round 1	Round 2	Round 1	Round 2
Accuracy								
Observer 1	54.5	66.7	22.2	41.2	81.8	81.8	81.8	81.8
Observer 2	83.3	77.3	64.3	53.8	93.9	86.4	95.5	90.9
Observer 3	65.2	75.8	66.7	30.8	86.4	90.9	87.9	90.9
Observer 4	72.7	68.2	64.7	64.7	90.9	93.9	90.9	93.9
Observer 5	68.2	63.6	52.9	41.2	93.9	93.9	95.5	95.5
Sensitivity								
Observer 1	23.5	41.2	10.0	18.2	67.6	67.6	76.5	79.4
Observer 2	67.6	58.8	62.5	44.4	88.2	76.5	97.1	97.1
Observer 3	41.2	52.9	75.0	0.0	82.4	82.4	91.2	94.1
Observer 4	47.1	38.2	60.0	54.5	82.4	88.2	88.2	97.1
Observer 5	41.2	29.4	54.5	33.3	91.2	91.2	97.1	97.1
Specificity								
Observer 1	87.5	93.8	37.5	83.3	96.9	96.9	87.5	84.4
Observer 2	100.0	96.9	66.7	75.0	100.0	96.9	93.8	84.4
Observer 3	90.1	100.0	57.1	66.7	90.6	100.0	84.4	87.5
Observer 4	100.0	100.0	71.4	83.3	100.0	100.0	93.8	90.6
Observer 5	96.9	100.0	50.0	60.0	96.9	96.9	93.8	93.8
Positive predictive value								
Observer 1	66.7	87.5	16.7	66.7	95.8	95.8	86.7	84.4
Observer 2	100.0	95.2	71.4	80.0	100.0	96.3	94.3	86.8
Observer 3	82.4	100.0	66.7	0.0	90.3	100.0	86.1	88.9
Observer 4	100.0	100.0	75.0	85.7	100.0	100.0	93.8	91.7
Observer 5	93.3	100.0	66.7	66.7	96.9	96.9	94.3	94.3
Negative predictive value								
Observer 1	51.9	60.0	5.9	35.7	73.8	73.8	77.8	79.4
Observer 2	74.4	68.9	25.0	37.5	88.9	79.5	96.8	96.4
Observer 3	59.2	66.7	35.0	36.3	82.9	84.2	90.0	93.3
Observer 4	64.0	60.4	30.0	50.0	84.2	88.9	88.2	96.7
Observer 5	60.8	57.1	38.1	27.3	91.2	91.2	96.8	96.8

Data are %

When all 33 cases were included in the analysis, the mean Cohen’s kappa coefficient for intra-observer reliability for the Klein’s line was 0.611 (median 0.659), and for the modified Klein’s line, it was 0.645 (median 0.670), both showing substantial agreement. For the S-sign in the VD extended-leg position, the mean Cohen’s kappa coefficient for intra-observer reliability was 0.883 (median 1), and for the S-sign in the VD frog-leg position, it was 0.903 (median 0.909), both showing almost perfect agreement. The mean Cohen’s kappa coefficient for inter-observer reliability for the Klein’s line was 0.540 (median 0.551), showing moderate agreement; for the modified Klein’s line, it was 0.616 (median 0.600), showing substantial agreement; for the S-sign in the VD extended-leg position, it was 0.791 (median 0.756), showing substantial agreement; and for the S-sign in the VD frog-leg position, it was 0.845 (median 0.879), showing almost perfect agreement.

When the fully displaced FCE control group was excluded from analysis, the Klein’s line was able to identify SCFE with a mean accuracy of 60.6% (median 60.0%), sensitivity of 30.6% (median 29.7%), specificity of 97.9% (median 100.0%), positive predictive value of 93.0% (median 100.0%) and negative predictive value of 55.3% (median 54.6%). The modified Klein’s line was again only performed in unilateral cases that did not produce a positive result for the Klein’s line. This yielded a mean accuracy of 50.4% (median 53.4%), sensitivity of 40.2% (median 49.5%), specificity of 68.0% (median 66.7%), positive predictive value of 55.2% (median 66.7%) and negative predictive value of 34.0% (median 35.4%). The S-sign performed in the VD extended-leg position produced a mean accuracy of 88.8% (median 92.0%), sensitivity of 79.2% (median 84.6%), specificity of 99.2% (median 100.0%), positive predictive value of 99.2% (median 100.0%) and negative predictive value of 82.2% (median 85.7%). When the S-sign was performed in the VD frog-leg position, the mean accuracy was 92.4% (median 93.0%), sensitivity of 93.9% (median 96.2%), specificity of 90.9% (median 91.7%), positive predictive value of 91.8% (median 92.3%) and negative predictive value of 93.5% (median 95.6%). These results are summarised in Table 3. The mean results from each round of readings for each of the observers have also been provided in Table 4.

**Table 3:** The accuracy, sensitivity, specificity, positive predictive value and negative predictive value for the Klein’s line, modified Klein’s line, S-sign in the ventrodorsal (VD) extended-leg position and the S-sign in the VD frog-leg position when the fully displaced femoral capital epiphyses control group was excluded from the analysis

	Klein’s line	Modified Klein’s line	S-sign VD extended-leg	S-sign VD frog-leg
Accuracy (%)				
Mean	60.6	50.4	88.8	92.4
Median	60	53.4	92	93
Sensitivity (%)				
Mean	30.6	40.2	79.2	93.9
Median	29.7	49.5	84.6	96.2
Specificity (%)				
Mean	97.9	68	99.2	90.9
Median	100	66.7	100	91.7
Positive predictive value (%)				
Mean	93	55.2	99.2	91.8
Median	100	66.7	100	92.3
Negative predictive value (%)				
Mean	55.3	34	82.2	93.5
Median	54.6	35.4	85.7	95.6

**Table 4:** The mean results from each round of readings from each of the observers when the fully displaced femoral capital epiphyses control group was excluded from the analysis

	Klein’s line		Modified Klein’s line		S-sign extended		S-sign frog-leg	
	Round 1	Round 2	Round 1	Round 2	Round 1	Round 2	Round 1	Round 2
Accuracy								
Observer 1	50.0	58.0	29.4	35.7	80.0	80.0	86.0	90.0
Observer 2	80.0	50.0	64.3	53.8	92.0	86.0	94.0	92.0
Observer 3	62.0	70.0	66.7	30.8	94.0	88.0	94.0	92.0
Observer 4	64.0	58.0	64.7	64.7	92.0	92.0	96.0	92.0
Observer 5	62.0	52.0	52.9	41.2	92.0	92.0	94.0	94.0
Sensitivity								
Observer 1	11.5	28.6	18.2	0.0	61.5	61.5	80.8	88.5
Observer 2	61.5	46.2	62.5	44.4	84.6	73.1	96.2	96.2
Observer 3	30.8	42.3	75.0	0.0	88.5	76.9	96.2	96.2
Observer 4	30.8	19.2	60.0	54.5	84.6	84.6	96.2	96.2
Observer 5	26.9	7.7	54.5	33.3	88.5	88.5	96.2	96.2
Specificity								
Observer 1	91.7	95.5	50.0	100.0	100.0	100.0	91.7	91.7
Observer 2	100.0	95.8	66.7	75.0	100.0	100.0	91.7	87.5
Observer 3	95.8	100.0	57.1	66.7	100.0	100.0	91.7	87.5
Observer 4	100.0	100.0	71.4	83.3	100.0	100.0	95.8	87.5
Observer 5	100.0	100.0	50.0	60.0	95.8	95.8	91.7	91.7
Positive predictive value								
Observer 1	60.0	88.9	40.0	0.0	100.0	100.0	91.3	92.0
Observer 2	100.0	92.3	71.4	80.0	100.0	100.0	92.6	89.3
Observer 3	88.9	100.0	66.7	0.0	100.0	100.0	92.6	89.3
Observer 4	100.0	100.0	75.0	85.7	100.0	100.0	96.2	89.3
Observer 5	100.0	100.0	66.7	66.7	95.8	95.8	92.6	92.6
Negative predictive value								
Observer 1	48.9	51.2	25.0	35.7	70.6	70.6	81.5	88.0
Observer 2	70.6	48.9	25.0	37.5	85.7	77.4	95.7	95.5
Observer 3	56.1	61.5	35.0	36.3	88.9	80.0	95.7	95.5
Observer 4	57.1	53.3	30.0	50.0	85.7	85.7	95.8	95.5
Observer 5	55.8	50.0	38.1	27.3	88.5	88.5	95.7	95.7

Data are %

When the fully displaced FCE control group was excluded from analysis, the mean Cohen’s kappa coefficient for intra-observer reliability for the Klein’s line was 0.305 (median 0.336), and for the modified Klein’s line, it was 0.572 (median 0.570), showing fair and moderate agreement, respectively. For the S-sign in the VD extended-leg position, the mean Cohen’s kappa coefficient for intra-observer reliability was 0.817 (median 0.839), and for the S-sign in the VD frog-leg position, it was 0.924 (median 0.920), both showing almost perfect agreement. The mean Cohen’s kappa coefficient for inter-observer reliability for the Klein’s line was 0.411 (median 0.510), and for the modified Klein’s line, it was 0.603 (median 0.584), both showing moderate agreement; for the S-sign in the VD extended-leg position, it was 0.912 (median 1.000), and for the S-sign in the VD frog-leg position, it was 0.936 (median 0.960), both showing almost perfect agreement.

## Discussion

The results of this study support our hypothesis that the S-sign is more effective than the Klein’s line and modified Klein’s line for the diagnosis of SCFE in cats. Excluding cases of fully displaced FCE, the S-sign on the VD frog-leg radiographs more accurately diagnosed SCFE than the S-sign on the VD extended-leg radiographs and the Klein’s line (92.4% vs 88.8% vs 60.6%, respectively), and had the greatest sensitivity (93.9% vs 79.2% vs 30.6%, respectively). The S-sign on the VD extended-leg radiographs had greater specificity than the Klein’s line and S-sign on the VD frog-leg radiographs (99.2% vs 97.9% vs 90.9%, respectively). The modified Klein’s line detected SCFE in 40.2% of cases that were negative for Klein’s line. In addition, intra-observer and inter-observer reliabilities were greatest for the S-sign in both VD extended-leg and frog-leg views with almost perfect agreements. In comparison, intra- and inter-observer reliabilities for the Klein’s line were fair and moderate, respectively, and were both moderate for the modified Klein’s line.

The S-sign in the frog-leg lateral pelvic view in humans has produced very favourable results for the diagnosis of SCFE, with an accuracy, sensitivity and specificity of 92.4%, 89.0% and 95.2%, respectively.^19^ For the purpose of this study, we analysed the S-sign in both VD extended-leg and VD frog-leg views. For both views, our results when the fully displaced FCE cases were excluded are similar to those found in this human study. It is also useful to find good results with this method in the VD extended-leg view and this will be particularly useful in cases where only one of these radiographic views is available for interpretation.

Excluding the fully displaced FCE control group, the accuracy and sensitivity of the Klein’s line in the present study were lower than those reported in the human literature at 60.6% and 30.6%, respectively, but specificity was higher at 97.9%. The Klein’s line has shown variable success in the identification of SCFE in humans. Two studies found it had poor sensitivity, with one study reporting a sensitivity of 39% from 23 cases,^17^ and another reporting a sensitivity of 40.3% from 30 cases.^18^ A more recent study found greater success with the Klein’s line, with an accuracy of 79.2%, sensitivity of 68.3% and specificity of 89.0% across 35 patients. In the human literature, it has been described that the FCE initially displaces posteriorly before it then displaces medially, which is why epiphyseal displacement is often missed on an AP pelvic radiograph and is why the Klein’s line has poor sensitivity as it will only detect medial displacement.^16,17^ It is possible that this same pattern and progression of FCE displacement occurs in cats.

In our study, the modified Klein’s line produced a sensitivity of 40.2%, but it must be appreciated that any cases with positive Klein’s line results, or that were bilaterally affected, were not included in the analysis of this method. Therefore, it is more accurate to consider that the modified Klein’s line in this study identified SCFE in 40.2% of the unilaterally affected cases that were not diagnosed via the Klein’s line method. The modified Klein’s line was developed to improve the sensitivity of the Klein’s Line.^18^ In comparison to the Klein’s line, the human literature reports that the modified Klein’s line has shown an improvement in sensitivity from 40.3% to 79.0%,^18^ and from 39% to 87%.^19^ Due to the differences in size between humans and feline patients, the difference of >2 mm required for a positive in humans was too high. Instead, we decided to use a difference of ⩾0.5 mm. Further research could be carried out to determine whether this value is appropriate, or whether a better value exists in order to achieve an optimum balance between sensitivity and specificity. It is important to appreciate that using the Klein’s line or modified Klein’s line methods alone may potentially miss a bilateral SCFE presentation. As an example, for the bilateral cases in this study, the Klein’s line only detected 41.7% of positive hips in round 1 and 30% in round 2. The modified Klein’s line requires one hip to be normal, and so use of this test in bilateral cases is inappropriate. However, you may not know whether a case is unilaterally or bilaterally affected, which limits the usefulness of this test.

Various definitions of the Klein’s line exist in the human literature. It is most commonly described as a line drawn along the superior margin of the femoral neck on an AP radiograph.^11,18,19^ This definition cannot be applied to our cat population because the superior margin of the femoral neck in humans is much straighter than the much more concave superior margin of the femoral neck in cats. An alternative definition in human literature defines the Klein’s line as the tangent to the concavity on the superior margin of the femoral neck.^16,21^ Owing to the concavity that exists in our feline population, we chose to adopt the latter definition in this study. This concavity may explain why the intra- and inter-observer reliabilities were lower for the Klein’s line and modified Klein’s line than the other methods, because there may be different interpretations of where the proximal and distal points of the lateral femoral neck lie. In addition, appreciation of where the tangent lies on a non-perfect curvature is likely to vary among observers. Periosteal reactions to the femoral neck can also obscure determination of where the tangent should lie.

There are several limitations to this study. The sample size is small and so a greater number of cases could be analysed in the future, preferably where each diagnostic method is tested on a large number of mildly affected cases, as this is when these diagnostic tools will be most useful. The determination of the position of the Klein’s line is likely to have varied between observers. The decision to use 0.5 mm for the modified Klein’s line test was not based on any scientific testing, and so further research could also be directed to finding the most accurate value to use. The quality of patient positioning for the radiographs also varied between cases, with pelvic rotation, degree of hindlimb rotation, and angle of flexion and extension differing among cases.

## Conclusions

This is the first published report describing the use of the Klein’s line, modified Klein’s line and S-sign to detect SCFE in cats. The S-sign in both VD extended-leg and VD frog-leg views produced results with high reliability, sensitivity, specificity, and positive and negative predictive values for detection of SCFE in cats. The S-sign can be used to increase early diagnosis and treatment in cats with SCFE that have only subtle radiographic changes. The use of the Klein’s line to detect SCFE in cats would not be recommended by the authors. The modified Klein’s line did show some merit by detecting 40% of SCFE cases that were not detected via the Klein’s line, but caution must be taken to avoid missing bilateral cases.
